# Assessment accommodations for autistic learners in South African schools: Stakeholder perspectives

**DOI:** 10.4102/ajod.v15i0.1803

**Published:** 2026-02-28

**Authors:** Yvonne Nell, Alta Kritzinger, Marien A. Graham, Renata Eccles

**Affiliations:** 1 Department of Speech-Language Pathology and Audiology, Faculty of Humanities, University of Pretoria, Pretoria, South Africa; 2 Department of Mathematics Education, College of Education, University of South Africa, Pretoria, South Africa

**Keywords:** inclusive education, autism spectrum disorders, assessment accommodations, primary school, high school, stakeholders

## Abstract

**Background:**

Autistic learners benefit from demonstrating academic knowledge with the help of assessment accommodations, guided by South African examination policies, such as the *National Policy Pertaining to the Conduct, Administration and Management of Examinations and Assessment for the National Senior Certificate Examination*. However, stakeholder perspectives on accommodations remain under-explored.

**Objectives:**

This study explored stakeholder perspectives (autistic adults, caregivers, educators, psychologists, speech-language therapists and occupational therapists) on assessment accommodations for autistic learners in South African schools.

**Method:**

A web-based questionnaire was distributed nationally to professionals and caregivers (*n* = 92). Quantitative data were analysed descriptively, and qualitative responses were thematically coded.

**Results:**

Stakeholders reported a persistent policy-practice disconnect, with educators lacking the knowledge of accommodation policies, as well as the training to implement accommodations, particularly for autistic learners. Similarly, current policies do not adequately accommodate the needs related to sensory regulation and anxiety. Considerable variability emerged in accommodation preferences, reflecting both the heterogeneity of autistic learners and the differences across stakeholder groups. Respondents also prioritised universal design elements such as simplified language, redundancy and clearer assessment layouts, which are not currently considered in South African policy. Overall, findings highlight the need for expanded and individually tailored assessment accommodations informed by diverse stakeholder input.

**Conclusion:**

The findings highlight a disconnect between policy and practice. Broader autism-specific accommodations are crucial to support equitable assessment conditions in South African schools, especially for learners with sensory and communication challenges.

**Contribution:**

This study provides insight into stakeholder experiences and suggests that current assessment policies may inadvertently exclude autistic learners. The findings support the need for inclusive, contextually relevant assessment strategies. The contribution aligns with the focus of the journal on disability inclusion by advancing evidence-based recommendations that promote full participation of neurodivergent learners in education systems, particularly within under-resourced and diverse settings.

## Introduction

A persistent challenge within inclusive education is that, although designed to promote fair and valid assessment, the assessment policies do not always effectively support the range of barriers experienced by learners with learning difficulties (Douglas et al. 2016). Despite South Africa’s policy frameworks aiming to ensure equitable assessment for all children, educators still experience difficulty in translating these guidelines into practice (Keen, Webster & Ridley 2016). This policy-practice disconnect is especially pertinent for autistic learners, who frequently experience barriers – such as sensory processing differences, anxiety, executive functioning challenges and communication difficulties – that are inadequately addressed by the assessment accommodations currently offered in South African policy (Ashburner, Ziviani & Rodger 2010; Den Houting et al. 2019; Lindsay et al. 2014). As autism prevalence increases, and with it the number of autistic learners in mainstream settings, reviewing the current practices for assessment accommodation has become increasingly important (Anderson, Smith & Iovannone 2018; Hodges et al. 2020). Such reviews should explore stakeholder perspectives, as these persons are central to recommending and implementing accommodations (Happé & Frith 2020; Pellicano, Dinsmore & Charman 2014).

Educators, as stakeholders, often struggle to manage the barriers experienced by autistic learners in assessment (Erasmus, Kritzinger & Van der Linde 2019; Witmer & Ferreri 2014). South Africa’s inclusive education system is guided by a social model of disability and emphasises participation and access for all learners (Nel et al. 2016). This approach is outlined in policy documents such as ‘Education White Paper 6’ (DoE 2001) and the ‘Policy on Screening, Identification, Assessment and Support’ (Department of Basic Education [DBE] 2014a). For assessment accommodations, these policies are supported by Annexure C1 of the *National Policy Pertaining to the Conduct, Administration and Management of the National Senior Certificate Examination* (DBE 2014b), which establishes eligibility and outlines the accommodations available to learners with barriers to assessment. Although autistic learners are included within the broader disability categories in these policies, the provided accommodations do not fully address the diverse and often nonacademic barriers that affect their assessment performance. Challenges with anxiety, sensory regulation, metacognition, executive functioning and language remain insufficiently supported within current policy frameworks (Ashburner et al. 2010; Den Houting et al. 2019; Lindsay et al. 2014).

Educators and other stakeholders attempt to mitigate these barriers by recommending and implementing the available accommodations according to policy (Larson et al. 2020; Wilkinson & Twist 2010). Assessment accommodations must be responsive to the barriers experienced by learners to prepare and support them effectively in assessment (Songlee et al. 2008; Tyrrell & Woods 2018). Yet the effectiveness of these measures depends on their relevance to each learner’s needs and on the training and confidence of those applying them (Larson et al. 2020; Wilkinson & Twist 2010). Evidence suggests that learners, particularly those with autism, may benefit from specific preparatory supports, such as direct training or structured familiarisation, to complement the accommodations and reduce assessment-related stress (Songlee et al. 2008; Tyrrell & Woods 2018). Given that summative assessments in South Africa occur annually from Grade 4 onwards (DBE 2012), the need for effective and developmentally appropriate accommodations is continuous throughout schooling.

According to national policy, the management of barriers to learning and assessment relies on multistakeholder collaboration (DBE 2014a). Speech-language therapists and allied health professionals work alongside educators to support learners and contribute to the translation of policy into practice (Wium & Louw 2013). Stakeholders provide insight for the translation of educational policy to practice (Roberts & Simpson 2016), and conversely, stakeholder practices can inform policy (Lord 2010). Participatory research in autism emphasises the importance of incorporating the perspectives of the community, educator and clinician to ensure that recommendations reflect real-world contexts, responding to the needs of autistic learners and translating these insights into effective policy (Pellicano 2020; Saggers et al. 2019).

Given the increasing recognition of diverse learning needs, assessment policies should respond to the varied and complex profiles of autistic learners. Internationally, universal design for learning (UDL) has been proposed to enhance flexibility in teaching, learning and assessment (Brown & Coomes 2016; Wood & Happé 2020), thereby reducing reliance on retrospective, individualised accommodations (Capp 2020; O’Neill & Padden 2022). Although White Paper 6 emphasises flexible curriculum delivery (DoE 2001), practical application within South African assessment structures remains limited. Autism has been identified as an important basis for developing universal design approaches, given the wide-ranging differences in communication, executive functioning and sensory processing among autistic learners (Burgstahler & Russo-Gleicher 2015; Sarrett 2018).

Exploring stakeholder perspectives on assessment accommodations for autistic learners may be a valuable starting point for refining policy and potentially integrating universal design principles (Saggers et al. 2019; Wood & Happé 2020). Such perspectives are particularly important in South Africa, where autism prevalence in schools is increasing (Pillay et al. 2021) and where the unique needs of autistic learners require more nuanced and responsive accommodations (Lindsay et al. 2014). The aim of this study, which formed a part of a study in fulfilment of the requirement for the degree of MA Speech-Language Pathology (Nell 2022), was therefore to describe the perspectives of stakeholders on various aspects of assessment accommodations as they relate to autistic learners in Grades 4–12 within South African schools (Nell 2022).

## Research methods and design

A mixed-method survey design was used (Nell 2022). Stakeholders’ perspectives were collected through a researcher-designed, self-administered, web-based questionnaire distributed by Qualtrics software (Version: June 2021).

### Respondents

Stakeholder groups included autistic adults, caregivers of autistic persons, psychologists, speech-language therapists (SLTs) and occupational therapists (OTs). Caregivers of autistic persons who had completed at least Grade 4 of a mainstream curriculum qualified for inclusion, as did autistic adults who had completed at least Grade 4 in South Africa. The criteria for selection to professional groups (educators, psychologists, SLTs and OTs) included experience with autistic learners (Grades 4–12) or experience in adjudication of assessment accommodations at district or provincial education offices. All respondents needed to be over 18 years of age and be sufficiently proficient in the English language to complete the questionnaire.

One hundred and eighteen individuals consented to participate. However, 16 questionnaires were largely incomplete and were discarded, leaving 102 respondents and demonstrating an 86.4% completion rate. Respondents had to self-identify their stakeholder groups. A difficult decision was made to exclude 10 individuals identified as belonging to two stakeholder groups. Excluding these 10 participants was considered necessary as one aim of the study was to compare different stakeholder groups, using the two-proportion *z*-test for independent groups. Including these respondents in both of the stakeholder groups they identified would result in a small percentage of the sample being dependent observations, invalidating the independent groups. Additionally, such a move would over-represent the voice of these 10 participants as their responses would be included in multiple groups. The authors acknowledge that these hybrid groups can offer valuable insights that bridge personal and professional experiences, highlighting the complexities of educational support. The final sample size was therefore 92 respondents.

Using G*Power software (Faul et al. 2007), we calculated the minimum sample size required for a two-proportion *z*-test at a significance level of 0.05, with a statistical power of at least 0.80 and a medium effect size. The analysis indicated that a minimum of 64 responses was necessary. These parameters were chosen because detecting medium effects with adequate power is generally considered sufficient for meaningful interpretation, whereas minimal effects may reach statistical significance (e.g. *p* < 0.05), although often lacking practical relevance (Peeters 2016). The achieved sample size of 92 participants exceeded the recommended minimum, ensuring that the statistical tests had the required power.

When respondents selected ‘other’ when qualifying their involvement in autism, they were included in the stakeholder group closest to their stated involvement: ‘Child psychiatrist’ and ‘social worker’ were grouped with psychologists and ‘sibling with autism’ was grouped with caregivers. The final sample had four autistic adults (*n* = 4/92; 4.3%), 14 caregivers (*n* = 14/92; 15.2%), 15 educators (*n* = 15/92; 16.3%), 27 OTs (*n* = 27/92; 29.3%), 10 psychologists (*n* = 10/92; 10.9%), 18 SLTs (*n* = 18/92; 19.6%), and three ‘other’ (*n* = 3/92; 3.3%) responses, in which the involvement was not qualified, and one respondent did not select a group (*n* = 1/92; 1.1%).

The majority of respondents were English-speaking (*n* = 59/92; 64.1%), from Gauteng (*n* = 34/92; 37.0%) and the Western Cape provinces (*n* = 24/92; 28.3%), the two most densely populated provinces within South Africa. Many respondents were noted to work in more than one setting, but predominantly in private practice (25/70; 35.7%), private centre or school (23/70; 32.9%) and state special school (15/70; 21.4%). Almost a third (19/70; 27.1%) of the respondents in the professional groups (educators, psychologists, SLTs and OTs) had more than 10 years’ experience with autistic learners; 22.9% (16/70) had 6–9 years’ experience; 22.9% (16/70) had 3–5 years’ experience; 21.4% (15/70) had less than 3 years’ experience and 5.7% (4/70) did not indicate their experience level.

### Material

Questions in the questionnaire were compiled by reviewing policy documents related to assessment accommodations within South Africa, including Annexure C1 of the *National Policy Pertaining to the Conduct, Administration and Management of the National Senior Certificate Examination* (DBE 2014b; DoE 2001). The table summarising accommodations from this document was included in the questionnaire for participant perusal. Additionally, the literature referencing accommodations for various barriers to assessment was consulted (Elliot, Kratochwill & Schulte 1999; Larson et al. 2020). Autism-associated characteristics that could impact assessment performance at the school level were considered (Ashburner et al. 2010; Tamm et al. 2020).

The questionnaire, designed for completion within 15–20 min, consisted of dichotomous, nominal multiple-choice and Likert-type questions that were closed-ended, as well as open-ended questions to obtain rich in-depth information on the elicited perspectives.

### Procedures

A mixed-method survey design was used. Stakeholders’ perspectives were collected through a researcher-designed, self-administered, web-based questionnaire distributed by Qualtrics software (Version: June 2021). Links to the web-based questionnaire were posted on the Facebook™ pages of South African professional organisations and South African therapist groups, teacher groups and autism groups. All had granted permission to have the link shared on their pages. When the link was clicked, the informed consent letter was displayed, requiring consent before proceeding to the questionnaire. Organisations (South African Speech-Language-Hearing Association, Occupational Therapy Association of South Africa, South African Association for Child and Adolescent Psychiatry and Allied Professions, and Autism South Africa) were also approached to distribute the link to their member databases.

### Data analysis

All data were analysed by using SPSS Statistics software, version 27. The two-proportion *z*-test was used to make pair-wise comparisons among stakeholder groups. A *p*-value less than 0.05 indicated that the responses differ significantly. The 5-point Likert scales were collapsed into three categories: disagreement, neutrality and agreement.

Qualitative information gathered from open-ended questions was initially coded by the first author, and a consensus of themes was then achieved among the authors. In the qualitative component, thematic saturation was approached as no substantially new themes emerged after coding the final set of responses. However, we acknowledge that the limited representation of educators and autistic adults may have constrained the diversity of perspectives.

### Ethical considerations

Ethical clearance to conduct this study was obtained from the Research Ethics Committee of the Faculty of Humanities, University of Pretoria (No. HUM022/0720).

## Results

Despite having experience with autistic learners or being involved in adjudicating assessment accommodations, 63.0% (*n* = 58/92) of the respondents were not aware of Annexure C1 (DBE 2014b), as the guiding document for selecting and implementing assessment accommodations in South Africa. This document combines barriers to assessment related to ‘behaviour, anxiety, attention deficit/hyperactivity disorder (ADHD), autism and other psycho-social disorders’. When asked, approximately half of the respondents who answered the question (49.1%, *n* = 30/61) found the grouping of conditions inappropriate. A total of 46.6% (*n* = 34/73) of the respondents who responded to the question felt that the accommodations summarised in Annexure C1 (DBE 2014b) met the needs of autistic learners during assessment. The majority of respondents (80.3%; *n* = 61/76) to the question, however, indicated that more options for assessment accommodations should be allowed for autistic learners.

Considering accommodations for barriers that do not have a clear academic outcome is also essential (Ashburner et al. 2010; Brown & Coomes 2016). Anxiety is commonly associated with autism (Dieckhaus et al. 2021). A total of 48.3% (*n* = 28/58) of respondents felt that the accommodation options in Annexure C1 (DBE 2014b) did not accommodate autistic learners’ needs regarding anxiety experienced during assessments. Just over half (*n* = 29/56; 51.8%) of the respondents indicated that the use of ‘additional time’ would alleviate the anxiety felt by autistic learners during assessments. Respondents across the sample, however, reported that changes to the environment would alleviate neither the anxiety (90.9%; *n* = 50/55) nor the difficulties in sensory regulation experienced by autistic learners (100.0%; *n* = 55/55). Additionally, two-thirds of the respondents (67.2%; *n* = 39/58) reported that the current options in Annexure C1 do not accommoate the sensory needs of autistic learners during assessments.

Most respondents (80.3%; *n* = 61/76 respondents) indicated that additional options for assessment accommodation should be allowed for autistic learners. Respondents were given multiple-choice responses to include several assessment accommodations. The variability in the options selected for assessment accommodation to be included for autistic learners (866 total selections) was large. These differences may stem from the unique and varied needs of specific autistic learners, as well as the diverse stakeholders involved with these learners (Keen et al. 2016; Witmer & Ferreri 2014) (Table 1).

**TABLE 1:** Selections by stakeholders to be included as assessment accommodations for autistic learners within South African schools.

Accommodation	Currently in Annexure C1 (yes/no)	Name in Annexe C1	Restrictions on use according to Annexe C1	Number of responses	% out of 866 responses	% out of 92 respondents
**Specialised settings**						
Specialised setting: Low arousal; special lighting and/or acoustics	No	-	-	52	6.0	56.5
Specialised setting: Playing of calming music to minimise distractions	No	-	-	34	3.9	37.0
**Special equipment**						
Use of noise buffers and/or headphones	No	-	-	51	5.9	55.4
Use of stimulation toys	No	-	-	28	3.2	30.4
Use of headphones with music	No	-	-	30	3.5	32.6
**In-person supports**						
Familiar administrator and/or invigilator	No	-	-	48	5.5	52.2
Encourager and/or motivator: May provide verbal encouragement of learner’s efforts. Encouraging words to sustain effort	No	-	-	32	3.7	34.8
**Language supports**						
All directions and prompts in simplified language	No	-	-	50	5.8	54.3
Simplified Language (Restate question with more appropriate vocabulary or define vocabulary)	Yes	Rephrasing	Only for deaf and/or hearing impaired and learners who are aphasic	43	5.0	46.7
Visual supports for language	No	-	-	42	4.8	45.7
Individual assistance with directions of assessment (including interpretation)	No	-	-	34	3.9	37.0
Colour-coding of instructions to emphasise steps	No	-	-	32	3.7	34.8
Keywords highlighted	No	-	-	27	3.1	29.3
**Timing and/or scheduling**						
No two examinations on the same day	No	-	-	44	5.1	47.8
**Learner allowances**						
Allowing learners to voice ideas while engaging with the question paper	No	-	-	42	4.8	45.7
**Redundancy**						
Redundancy (question presented auditorily and written)	Yes	Reader	Not for the grouping that includes autism	50	5.8	54.3
Oral-delivery: in-person (i.e., reader)	Yes	Reader	Not for the grouping that includes autism	39	4.5	42.4
Oral-delivery: recorded	Yes	Reader	Not for the grouping that includes autism	35	4.0	38.0
Oral-delivery: reading pen or text-to-voice technology	Yes	Computer voice to text and/or text-to-voice	Not for the grouping that includes autism	33	3.8	35.9
**Presentation supports**						
All questions starting on a new page	No	-	-	44	5.1	47.8
Enlarged print (18pt)	Yes	Enlarged print	Not for the grouping that includes autism	19	2.2	20.7
Coloured question paper	No	-	-	20	2.3	21.7
**Other and/or no options**						
Other accommodations	-	-	-	7	0.8	7.6
Other universal design options	-	-	-	2	0.2	2.2
None of the universal design options selected	-	-	-	1	0.1	1.1
None of the options selected for either accommodations or universal design options	-	-	-	27	3.1	29.3

**Total**	-	-	-	**866**	**100**	-

Note: Multiple response options. The total number of responses (866) is larger than the sample size (92), as the respondents could select more than one option.

Prominent additions selected by respondents included: ‘specialised setting (low arousal, special lighting and/or acoustics)’ (6.0% of all 866 selections), ‘use of noise buffers and/or headphones’ (5.9% of 866 selections), ‘all directions and prompts in simplified language’ (5.8% of 866 selections), ‘redundancy – questions presented auditorily and in written form’ (5.8% of 866 selections), ‘familiar administrator and/or invigilator’ (5.5% of 866 selections), ‘no two examination in the same day’; (5.1% of 866 selections), ‘all questions starting on a new page’ (5.1% of 866 selections) and ‘simplified language, including the restating of questions with more appropriate vocabulary or provision of definitions of vocabulary’ (5.0% of 866 selections).

**FIGURE 1:**
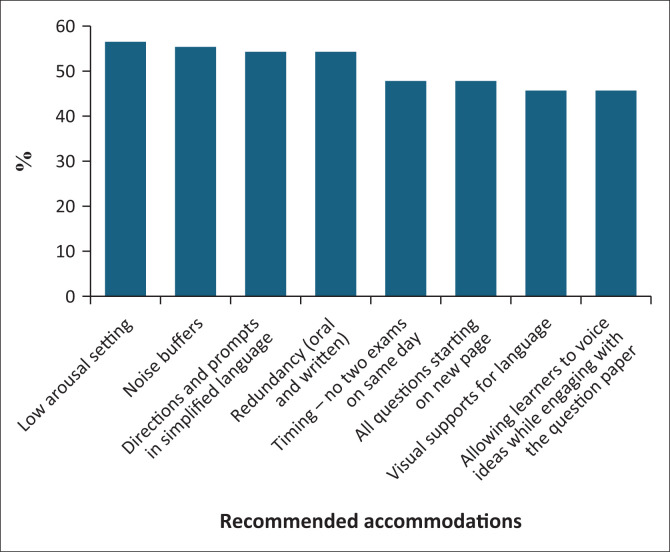
Most-selected accommodations recommended for autistic learners as a percentage of respondents (*N* = 92).

This information is perhaps easier to consider as a proportion of the total number of respondents selecting that particular option. (Figure 1).

Among stakeholder groups, caregivers selected accommodations for specialised settings significantly less than psychologists did (*z* = 1.706, *p* = 0.044). Likewise, the selections by psychologists for ‘no two examinations on the same day’ were significantly more compared to those by OTs (*z* = 1.685, *p* = 0.046). These differences appear to be related to perspectives of different professions, which necessitate the use of multistakeholder views. This finding was further supported by the given qualitative information. For example, one respondent noted:

‘1 paper per day. Sufficient time to study between exams and extra time to accommodate for sensory needs’ (Respondent 52, speech-language therapist, Gauteng)

Another respondent reiterated:

‘Short-notice rescheduling should be an option in the event of a panic attack or meltdown and/or shutdown (I believe it already is in the event of illness, so this is not a stretch in my opinion)’ (Respondent 17, autistic adult, Western Cape)

Selections by autistic adults (*z* = 2.226, *p* = 0.013) and caregivers (*z* = 1.787, *p* = 0.037) were significantly more compared to that by OTs for the use of an ‘encourager or motivator’ to provide ‘verbal encouragement of the learner’s efforts’.

In inclusive education settings, autistic learners may benefit from specific and direct training (as a pre-assessment accommodation) to help them manage the assessment environment (Songlee et al. 2008; Tyrrell & Woods 2018). Table 2 reveals that 20.8% of responses made for pre-assessment accommodations indicated that ‘teaching management of examination anxiety’ may help, whereas only 15.2% of the responses were for study guides and information about examinations (Table 2).

**TABLE 2:** Specific and direct training to be considered to support learners in preparation for assessment (pre-assessment accommodations).

Pre-assessment accommodations	Number of responses	% out of 289 responses†	% out of 92 respondents†	Responses to multiple response questions within the stakeholder category													
				Autistic adult		C/giver		Educator		Psych		SLT		OT		Other	
				*n*	%	*n*	%	*n*	%	*n*	%	*n*	%	*n*	%	*n*	%
Teaching how to manage examination anxiety	60	20.8	65.2	3	21.4	10	25.6	8	20.0	10	22.2	9	16.4	19	21.3	1	14.3
Teaching of test-taking skills	56	19.4	60.9	4	28.6	7	17.9	7	17.5	9	20.0	10	18.2	18	20.2	1	14.3
Pictures of examination invigilators and/or administrators	51	17.6	55.4	2	14.3	8	20.5	6	15.0	9	20.0	10	18.2	15	16.9	1	14.3
Information about examinations, why they are important and the provision of motivation in preparation for examinations	44	15.2	47.8	1	7.1	5	12.8	6	15.0	9	20.0	9	16.4	14	15.7	1	14.3
Study guide with practice questions	44	15.2	47.8	3	21.4	4	10.3	5	12.5	8	17.8	8	14.5	15	16.9	1	14.3
Other	5	1.7	5.4	1	7.1	1	2.6	2	5.0	0	0.0	1	1.8	0	0.0	0	0.0
None of the options selected	29	10.0	31.5	0	0.0	4	10.3	6	15.0	0	0.0	8	14.5	8	9.0	2	28.6

**Total**	**289**	**100.0**	-	**14**	**100.0**	**39**	**100.0**	**40**	**100.0**	**45**	**100.0**	**55**	**100.0**	**89**	**100.0**	**7**	**100.0**

c/giver, caregivers; psych, psychologists; SLT, speech-language therapist; OT, occupational therapist.

† , Multiple response options. The total number of responses (289) is larger than the sample size (92), as the respondents could select more than one option.

Again, the information from the remarks of individual respondents was rich, for example:

Time management, proficientcy in use of applicable support technology, modelling of exam routine (i.e. times to report, entering exam venue, handing out of papers, reading time, finishing off and leaving venues). Visual prompts should also be made avilable in the venues, with countdown timers as opposed to clocks. (Respondent 58, Educator, KwaZulu-Natal)

The venue where they write has to be familiar to the child; they have to be familiar with the administrator. They can’t write with other children they don’t know. They can’t write in noisy environments. Some can benefit from noise-cancellation earphones. They sometimes understand certain questions in the test completely wrong, or physically, the layout of the test can cause more anxiety. (Respondent 58, Educator, KwaZulu-Natal)

This view contrasts with another respondent:

Study guides will be useful for parents and tutors, but not to ASD [*Autism Spectrum Disorder*] learners themselves. They already have access to past papers, and too much variation causes confusion. (Respondent 35, parent, Western Cape)

The two-proportion *z*-test showed no significant differences among stakeholder groups for the information presented in Table 2.

Table 3 shows the differences among stakeholder groups in recommending how pre-assessment accommodations should be implemented (Table 3). Almost a third of the responses indicated that ‘educators should carry out specific and direct training for autistic learners as a matter of course’ (29.2%; *n* = 47/161). Almost one-fifth (18.6%; *n* = 30/161) of responses were for ‘a preapproved programme that offers specific and direct training by education districts’. The two-proportion *z*-test showed no significant differences among the responses of the different stakeholder groups, as presented in Table 3.

**TABLE 3:** Implementation of the pre-accommodations programme.

How should training be administered?	Number of responses	% out of 161 responses	% out of 92 respondents	Responses to multiple response questions within the stakeholder category													
				Autistic adult		C/giver†		Educator		Psych‡		SLT§		OT¶		Other	
				*n*	%	*n*	%	*n*	%	*n*	%	*n*	%	*n*	%	*n*	%
Educators should provide such programmes for autistic learners as a matter of course	47	29.2	51.1	3	27.3	8	36.4	8	36.4	8	36.4	6	17.6	13	28.9	1	25
Should be a specific training that has been approved by Province or District Education Offices and run in accordance with specific guidelines	30	18.6	32.6	3	27.3	3	13.6	2	9.1	5	22.7	8	23.5	8	17.8	1	25
Should be included as an accommodation, and thus approved by the District and/or Provincial accommodations committees	28	17.4	30.4	2	18.2	2	9.1	2	9.1	4	18.2	9	26.5	9	20	0	0
No application necessary for such pre-assessment accommodations	13	8.1	14.1	2	18.2	2	9.1	1	4.5	3	13.6	0	0.0	5	11.1	0	0
Educators should only provide such programmes for autistic learners should they feel it necessary	9	5.6	9.8	0	0.0	1	4.5	3	13.6	2	9.1	2	5.9	1	2.2	0	0
Such pre-assessment accommodations should not need to be documented	4	2.5	4.3	1	9.1	1	4.5	1	4.5	0	0.0	0	0.0	1	2.2	0	0
Such pre-assessment accommodations should not be provided unless requested, and then they should be approved by Province or District	1	0.6	1.1	0	0.0	0	0.0	0	0.0	0	0.0	1	2.9	0	0.0	0	0
No options selected	29	18.0	31.5	0	0.0	5	22.7	5	22.7	0	0.0	8	23.5	8	17.8	2	50

**Total**	**161**	**100.0**	**175.0**	**11**	**100.0**	**22**	**100.0**	**22**	**100.0**	**22**	**100.0**	**34**	**100.0**	**45**	**100.0**	**4**	**100**

Note: Multiple response options. The total number of responses (161) is larger than the sample size (92), as the respondents could select more than one option.

† , c/giver = caregivers;

‡ , psych = psychologists;

§ , SLT = speech-language therapist;

¶ , OT = occupational therapist.

## Discussion

The findings from this South African study have contributed to the body of research on assessment accommodations for autistic learners by describing the perspectives of different stakeholders involved in the education of autistic learners and those of autistic persons themselves. Stakeholder perspectives are important in autism research as they reflect on the practical and immediate concerns of the community (Pellicano 2020; Saggers et al. 2019).

The discussion will revolve around four main areas for consideration: policy-practice disconnect, the need for sensory and emotional supports, stakeholder variability, and implications for universal design in learning and future policy. Then, the strengths and limitations of the study will be explored while directing future research.

### Policy-practice disconnect

Findings from this study concur with previously documented inconsistencies in educator knowledge regarding assessment accommodations (Hodgson, Lazarus & Thurlow 2011). This finding was reiterated by one of the respondents’ comments:

‘All educators/staff involved with the ASD learner MUST BE TRAINED ON UNIVERSITY LEVEL to ensure the child and ASD is understood. Educators without proper training will not be able to support these learners effectively. Special Needs training for educators is extremely important and not attended to.’ (Respondent 2, educator, Gauteng)

Autistic learners are therefore at risk, as educators and other support staff may not be aware of the support that can be offered, as documented in policy. Similarly, a lack of knowledge of education policies hinders lobbyists for change and the translation of policy to practice. Consequently, training and collaboration are needed regarding assessment accommodations for autistic learners across inclusive and special schools (Larson et al. 2020; Wilkinson & Twist 2010). This policy is vitally important as the incidence of autism and the enrolment of autistic learners are rising, as shown by studies in the Western Cape province of South Africa and elsewhere in the world (Lindsay et al. 2014; Pillay et al. 2021, 2022).

Almost half of the respondents felt that the accommodations summarised in Annexure C1 (DBE 2014b) accommodated the needs of autistic learners during assessment, and yet the majority indicated that additional accommodations would be valuable. This view may be related to the fact that respondents felt that existing accommodations have a clear academic focus but do not support the barriers affecting participation in assessments, such as anxiety and sensory regulation needs.

This South African study has demonstrated the need for collaboration and training on assessment accommodations across the education system and for relevant stakeholders to adequately support autistic learners. Policy implementation should evolve, and research findings, such as this study, should inform policy and practice (Lazarus et al. 2009).

### The need for sensory and emotional supports

Previous research has indicated a need to recognise the ‘more individualised, unique and autism-specific needs’ of autistic learners (Saggers et al. 2019), rather than grouping autism with other conditions. Respondents emphasised that autistic learners should be considered individuals with different needs and that assessment accommodations must reflect this view:

‘I feel we need to stop trying to form blanket options for learners and rather see them as individuals with different needs. Our responsibility is to help them to achieve according to their full potential, not to try and catch them out.’ (Respondent 66, remedial educator, Eastern Cape)

‘There is no global policy with regard to ASD assessment supports. The needs of the student must be determined holistically via psych-ed assessments, observations, examinations leading up to the National Senior Certificate (NSC) exams and a range of assessment methods to determine best practices for each individual.’ (Respondent 58, educator, KwaZulu-Natal)

Respondents noted that current accommodations do not sufficiently support barriers related to anxiety and sensory regulation. In the United States, for example, over 90% of postsecondary institutions used academically focused accommodations, but only a third provided sensory accommodations (Brown & Coomes 2016). Global research has indicated the need to address sensory and other nonacademic support for autistic learners in both classroom and assessment settings (Ashburner et al. 2010; Brown & Coomes 2016).

The most-selected additional assessment accommodation in this study was ‘specialised settings: low arousal; special lighting and/or acoustics’, despite respondents simultaneously indicating uncertainty about the effectiveness of environmental changes for reducing anxiety. Sensory-friendly environments as assessment accommodations are not widely studies in the literature (Lai & Berkley 2012; Leifler et al. 2021). However, at least one study demonstrated that low-distration environments can positively affect concentration for students with sensory defensiveness (Lewis & Nolan 2013), and the autism community continues to advocate for sensory-related accommodations (Sarrett 2018). Respondent comments further highlighted the need for sensory-friendly environments and personalised regulatory supports:

‘Due to the highly variable nature of ASD, I believe each learner’s environment should be optimised. Some may require regular intervals, movement and/or other regulatory activities.’ (Respondent 90, speech-language therapist, Western Cape)

‘Permission to wear comfortable clothing even if against school uniform policy to avoid sensory overstimulation.’ (Respondent 17, autistic adult, Western Cape)

Provision of additional time has been recommended as reasonable accommodation for exam stress and anxiety (Jansen et al. 2017). Just over half of the respondents agreed that additional time would alleviate anxiety for autistic learners.

The pre-assessment training suggestions by respondents are similar to previous (Simpson, Griswold & Smith Myles 1999; Songlee et al. 2008; Tamm et al. 2020; Tyrrell & Woods 2018), showing that preparation strategies reduce anxiety and support executive functioning. One autistic respondent highlighted the need for materials that are not based solely on nonautistic assumptions, emphasising risks such as miscommunication or gaslighting:

‘The reason why I didn’t mark the first one [*information about examinations, why they are important as well as provision of motivation in preparation for examinations*] was because I thought that people may mess it up, by providing information which is logical to THEM, but which is illogical to an autistic person; and in the end it just leads to gaslighting…’ (Respondent 18, educator and autistic adult, Western Cape)

### Stakeholder variability

The variability in the options selected for assessment accommodation (866 total responses) was large. These differences may stem from the unique and varied needs of autistic learners as well as the diverse stakeholders with whom they interact (Keen et al. 2016; Witmer & Ferreri 2014). Accommodations should therefore be selected in a bespoke fashion to accommodate individual needs.

A small number of statistically significant differences emerged across stakeholder groups. Although limited in number, these differences are meaningful. Psychologists and caregivers differed significantly in their views on the value of specialised settings. This divergence likely reflects the distinct experiential and professional vantage points from which these groups understand learning environments. Caregivers routinely adapt home contexts to support daily functioning, often using intuitive strategies to minimise sensory and environmental barriers (Schiavone et al. 2018). Hence, they may be less exposed to the kinds of environmental constraints arising within classroom settings, in which sensory, social and organisational demands are less routinely modified. Psychologists, by contrast, are trained to identify how environmental stressors exacerbate emotional, behavioural and self-regulatory challenges, which may heighten their recognition of the potential pitfalls with stressors related to assessment. These divergent insights illustrate how varying degrees of proximity to educational environments and differences in professional frameworks shape stakeholders’ perspectives of what supports are most needed.

Autistic adults and caregivers selected the use of an encourager or motivator significantly more often than OTs did. Although specific literature supporting the use of motivators is limited, it is plausible that lived experience offers unique insight into the importance of emotional co-regulation and motivational support during demanding scholastic tasks. The comment from an autistic respondent in this study underscores the heterogeneity of autistic profiles and highlights the role that a regulation partner or motivational presence may play in reducing stress, enhancing task engagement and supporting performance. Research on autistic self-advocacy similarly notes that autistic individuals often emphasise relational and regulatory supports that may be under-recognised in clinical or educational frameworks (Milton 2019; Pellicano et al. 2014). Thus, different endorsement rates for encouragers and/or motivators may reflect the value of integrating lived expertise, which can illuminate needs that professionals may overlook.

Psychologists also selected ‘no two exams on one day’ more frequently than OTs did. This choice may relate to psychologists’ training in emotional and behavioural well-being, including their attention to factors such as cognitive load, anxiety, fatigue and stress dysregulation (Kellems et al. 2016). The assessment spacing aligns with principles of reducing cumulative stressors, especially for learners with heightened vulnerabilities in emotional or executive functioning. OTs, while knowledgeable about environmental and sensory barriers, may place comparatively less emphasis on emotional load across multiple assessments.

Taken together, these group differences illustrate the importance of incorporating diverse stakeholder perspectives into research on assessment accommodations. Each group brings distinct forms of knowledge (professional expertise, contextual familiarity or lived experience) that shape their perceptions of what supports are necessary. The differences observed in this study highlight the risk of relying too heavily on any single stakeholder group, as doing so may inadvertently privilege certain types of expertise over others. Multistakeholder engagement, therefore, remains essential for developing assessment practices that are equitable, ecologically valid and responsive to the wide variability within the autistic population.

### Implications for universal design for learning and future policy

Education White Paper 6 notes that the curriculum must be flexible enough to accommodate different learning needs and styles (DoE 2001). The study findings align with this, emphasising the need to expand the available assessment accommodations and ensure flexibility across the education system. Such flexibility supports learners with executive function, communication difficulties and sensory needs, consistent with UDL.

The frameworks for inclusive education around the world have called for UDL and assessment (Capp 2020; Dolan & Hall 2001), but as yet, the change in assessment practices has been limited (O’Neill & Padden 2022). Universal design elements prioritised by respondents in this study included: ‘all directions and prompts in simplified language’, ‘redundancy (questions presented auditorily and in written form)’ and ‘all questions starting on a new page’. These accommodations are not currently available within South African policy (DBE 2014b), except redundancy, which is offered to learners with specific learning disabilities related to reading and writing. Respondents also called for pre-assessment accommodations as a specific training approved by provincial or district education offices, to be provided as a matter of course. Including this option as an element of universal design may benefit all learners and offer flexibility from the planning stage, and not a later adaptation. The need for strengthening the education system to support autistic learners is also obvious (Pillay et al. 2022), as materials should be available for teachers to use in support of learners with barriers to learning, including autistic ones. The pre-assessment training suggestions by the respondents in this study correlate with those proposed in earlier research (Simpson et al. 1999) to support autistic learners in preparation for assessment; by reducing anxiety and supporting executive functioning, as one respondent mentioned “as in the principal of social stories” (Respondent 90, Speech-language therapist, Western Cape). These suggestions support learners in ways similar to social stories, bu reducing anxiety, increasing familiarity and supporting executive functioning (Songlee et al. 2008; Tamm et al. 2020; Tyrrell & Woods 2018). Respondents prioritised universal design elements such as simplified language in directions, redundancy (written and auditory formats), and the starting of questions on new pages. These are not yet included in South African policy (DBE 2014b), except redundancy, for specific learning disabilities. Respondents also recommended universal pre-assessment training, which could benefit all learners and embed flexibility from the planning stage.

### Recommendations from the current study

Although some limitations in this study have been highlighted, it is important to consider how this study, with future studies, could create concrete opportunities for policy development and expansion. Further training for educators on autism is needed as a result of the increasing numbers and lack of knowledge about the specific barriers facing autistic individuals. This approach will, in turn, develop classroom-based accommodations that all educators can employ. Policy-makers should also consider what universal design aspects can be ‘built-in’ to assessment accommodations processes, such as providing redundancy should a learner want this, or presentation of exam papers with keywords explained and questions presented on separate pages. The ‘allowable’ assessment accommodations should also be broadened, allowing educators to select individual accommodations to suit every learning barrier, including those perceived as ‘nonacademic barriers’ that clearly impact academic performance. The importance of policy-makers consulting with varied stakeholders in developing these accommodations is important, and likewise, the importance of transverse teams at the district and provincial level is emphasised.

### Strengths and limitations

The strength of this study was its incorporation of perspectives from a range of stakeholders. This type of participatory research is valued as it can lead to contextually appropriate suggestions for the easy translation of research into policy and practice, thereby maximising success (Pellicano 2020; Pillay et al. 2022; Saggers et al. 2019). The study findings can be used to support the assessment of policy evolution in line with Education White Paper 6 (DoE 2001) and the *Screening, Identification, Assessment and Support (SIAS) policy* (DBE 2014a) in support of autistic learners in assessment.

As a result of the recruitment strategy, which relied on distributing survey links through Facebook™ groups, a sampling bias is likely. Respondents were already engaged in autism-related online communities, which may have skewed the sample towards individuals with heightened interest, awareness or access to autism-related resources. Consequently, teachers from rural or under-resourced districts were under-represented. This factor aligns with the findings of the study, which show that most respondents were situated in urban areas and were predominantly English or Afrikaans first-language speakers. These limitations have implications for both the generalisability of the results and the equity of the study, as the perspectives of educators in linguistically diverse, rural or resource-constrained contexts may not be adequately reflected. Perspectives of stakeholders from varied geographic settings, languages, cultures and socio-economic settings are needed to gather a comprehensive view.

Similarly, autistic adults (4.3% of respondents), caregivers (15.2%), educators (16.3%) and psychologists (10.9%) were notably under-represented, while OTs formed the largest stakeholder group (29.3%). This imbalance has important implications, as it may skew the findings towards a predominantly clinical or therapeutic perspective, thereby limiting the extent to which the lived experiences of autistic adults and the day-to-day insights of caregivers and educators are reflected in the data. Consequently, the perspectives most relevant to learner- and family-centred approaches may not be fully captured.

### Future research opportunities

Future research should broaden stakeholder representation to ensure more balanced and inclusive multistakeholder perspectives, particularly by more deeply engaging autistic adults and caregivers whose lived experiences are central to learner- and family-centred practice. Studies should also examine assessment accommodations across both mainstream and special school settings, as well as among professionals with varying levels of experience, to understand how contextual and expertise-related factors shape accommodation practices.

Further work is needed to evaluate the effectiveness and differential benefits of specific accommodations for autistic learners, thereby contributing to evidence-based practice. Additionally, research should investigate the support needs of educators in implementing accommodations within everyday teaching and learning activities, as well as formal assessments.

Finally, the opportunity for developing and empirically testing universal design strategies in curriculum and assessment to more proactively meet the needs of autistic learners is immense.

## Conclusion

Results from this South African study provided insight from stakeholder practice to inform and motivate policy evolution. Currently, the policy for assessment accommodations does not appear to be well-translated into practice within South Africa. Therefore, awareness campaigns, collaboration and training on the available assessment accommodations are needed to support autistic learners during assessment. The results also support the need for expanding the offering of assessment accommodations for autistic learners. This policy would underpin the move from deficit-driven accommodations towards social inclusion and neurodiversity (Happé & Frith 2020; Sarrett 2018), which can be facilitated through universal design principles. The spectrum of characteristics unique to autistic learners can be used to build universal design for assessment accommodations. Changes to policy and practice are necessary to support the needs of the growing number of autistic learners in South African schools, and indeed all learners with communication, sensory and executive function difficulties. Therefore, through consultation with stakeholders, the DBE must initiate formal policy-revision committees tasked with evaluating and updating the assessment policies in light of the contemporary understanding of ASD. Such committees should prioritise the heterogeneous nature of ASD. Attending systematically to this variability would enable the identification of barriers to assessment, which have not previously been considered, as well as developing differentiated assessment accommodations that respond to a range of learning and assessment needs.

Moreover, the DBE bears responsibility not only for formulating policy but also for ensuring the effective preparation of educators. This activity includes comprehensive training on the implementation of assessment accommodations and on pedagogical responsiveness to diverse learner populations, including those with ASD. A national strategy for delivering high-quality, standardised professional development, particularly targeting educators in rural and under-resourced schools, is essential. Such training must equip educators with an implicit and explicit understanding of how ASD-related barriers, including heightened anxiety, executive functioning differences, and sensory processing difficulties, may affect both learning and assessment performance.

By embedding a sustained focus on neurodiversity within the professional development of teachers, educators will be better positioned to identify nonacademic barriers to assessment and to implement relevant, evidence-based accommodations for all learners. This policy, in turn, will enhance the equity of learning outcomes and promote inclusive assessment practices across the education system.

Looking forward, we believe that South Africa is well-positioned to assume a leadership role in developing assessment accommodation frameworks that reflect the extensive heterogeneity of autistic profiles. By coupling ASD-specific accommodations with universal design principles that benefit all learners, the DBE could set a benchmark for inclusive assessment reform. Such a forward-looking approach would not only strengthen national policy coherence but also contribute to an emerging international discourse on equitable assessment in diverse educational contexts.
